# Seismic footprints of shallow dyke propagation at Etna, Italy

**DOI:** 10.1038/srep11908

**Published:** 2015-07-15

**Authors:** Susanna Falsaperla, Marco Neri

**Affiliations:** 1 Istituto Nazionale di Geofisica e Vulcanologia, Sezione di Catania, Osservatorio Etneo, Piazza Roma 2, 95125, Catania, Italy

## Abstract

One of the key issues in forecasting volcanic eruptions is to detect signals that can track the propagation of dykes towards the surface. Continuous monitoring of active volcanoes helps significantly in achieving this goal. The seismic data presented here are unique, as they document surface faulting processes close (tens to a few hundred meters) to their source, namely the dyke tip. They originated nearby - and under - a seismic station that was subsequently destroyed by lava flows during eruptive activity at Etna volcano, Italy, in 2013. On February 20, a ~600 m-long and ~120 m wide NW-SE fracture field opened at an altitude between 2750 and 2900 m. The consequent rock dislocation caused the station to tilt and offset the seismic signal temporarily. Data acquisition continued until the arrival of the lava flow that led to the breakdown of the transmission system. Shallow ground fracturing and repeated low-frequency oscillations occurred during two stages in which the seismic signal underwent a maximum offset ~2.57 × 10^4^ nm/s. Bridging instrumental recordings, fieldwork and conceptual modelling, these data are interpreted as the seismic footprints of a magmatic dyke intrusion that moved at speed ~0.02 m/s (first stage) and 0.46 m/s (second stage).

Etna, located in southern Italy, is one of the most active volcanoes in the world in historical time. It is also one of the most highly monitored, with state-of-the-art networks of geophysical and geochemical sensors operating continuously and providing surveillance over a territory of more than 1180 km^2^ prone to seismic and volcanic activity^1^.

The seismic station EBEL was set up on Etna in late 2004, at Belvedere at an altitude of 2899 m (Fig. 1). It was one of the 45 seismic stations on the volcano edifice belonging to the permanent monitoring network run by Istituto Nazionale di Geofisica e Vulcanologia (INGV). It was equipped with Nanometrics 3-component Trillium (40 s cutoff period) seismometers. The signals were sampled at a frequency of 100 Hz and were transmitted to the data acquisition centre in Catania. The other seismic stations used in our analyses had the same technical characteristics; they were within a maximum distance of ~8 km from the summit craters, and at altitudes between 1600 m (ESPC) and 3050 m (ECPN) (Fig. 1).

During the more than 8-year operational period of EBEL, numerous effusive (2004-2005, 2006, 2008-2009, 2013) and explosive (2006, 2007, 2008, 2011, 2012, 2013) eruptions affected the summit of the volcano and the Valle del Bove (Fig. 1). EBEL was close to the active craters of Etna (~800 m from the New South East Crater, Fig. 1a) and, therefore, highly prone to breakdown in case of paroxysmal/effusive activity. By February 28 2013, a lava flow had finally covered the station during a lava fountain episode, the 6^th^ from the beginning of the year.

The last 11 operational days of EBEL offered us a wealth of field observations on ongoing eruptive and fracturing processes, giving us the rare opportunity to document these processes close to their source. We use the seismic signal recorded as these processes were underway to piece together what happened at this time.

## Recent activity at Etna

There is continuous activity at the summit craters of Etna. There is a constant release of gas and vapour, occasional Strombolian activity and impressive episodic high-rate paroxysmal eruptions. At times, Etna also erupts through fissures in its flanks that are mainly clustered along three rift zones, the so-called NE-, S- and W-rift, respectively^2^. Dykes feeding eruptive fissures stem radially from the central conduit or, more rarely, originate from independent batches of magma^3^,^4^,^5^.

The development of extensional fractures, faults, and grabens accompanied magma intrusions and the subsequent volcanic activity during numerous historical flank eruptions of Etna (e.g., 1809, 1989, 1991–1993, 2001, 2002-2003, 2004-2005, 2009). This especially happened when the eruptive systems were along the NE- and S-Rift^3^,^5^,^6^,^7^,^8^. In the years from 1809 to 2010, the length of the eruptive fractures varied from a few tens of meters up to ~9 km (∼2,3 km on average)^5^,^8^. The complete extension of these eruptive fractures covered time spans from tens to a few hundreds of hours^5^; the mean velocity of propagation was 0.05 m/s, encompassed between the minimum value of 0.003 m/s (in 2004) and the maximum of 0.21 m/s (in 2008)^5^,^7^. However, the velocity was up to ~1 m/s during the initial phase of the fracturing process, similar to that inferred at Krafla, Iceland^9^.

Extensional fractures, faults and grabens accompany magma intrusions also in other volcanoes, such as Kilauea, Hawaii^10^, and Stromboli, Italy (in 2002 and 2007)^11^,^12^ . However, this is not a rule. For example, the dyke-induced tensile stresses associated with the feeder dyke did not generate any new surface faults at Krafla, Iceland, in 1980^13^.

**Figure 1:**
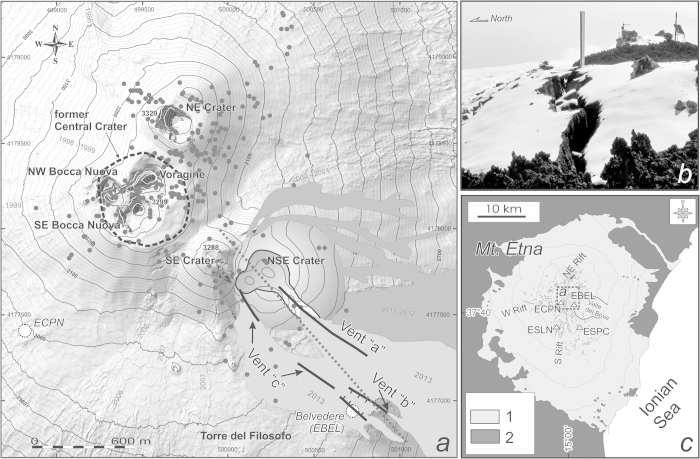
**a**) Fracture field (black lines), location of the active vents (Vent “a”, Vent “b”, Vent “c”) and centroid of volcanic tremor (solid grey circles) between 18 and 28 February 2013. The panel is a zoom of the area indicated by the dashed grey square in Fig. 1c. The NW-SE dashed grey line indicates the trace of the cross section in Fig. 4. The light grey lava flows in a) formed in 2013. The snapshot **b**) was taken by Michele Mammino on 22 February 2013, six days before the seismic station breakdown (EBEL is visible in the foreground). **c**) Map of Etna and location of the volcanic rifts (NE, W and S, respectively) and the seismic stations considered here (EBEL, ECPN, ESLN and ESPC). The map in a) was generated using a DEM owned by INGV; geographical coordinates are expressed in UTM projection, zone 33N.

There are five active craters at Etna’s summit: the Central Crater - CC (divided into Voragine – VOR, and Bocca Nuova - BN), the North-East Crater (NEC), the South-East Crater (SEC) and, since 2007, the youngest “New” South-East Crater^14^ (NSEC, Fig. 1a). The NSEC built up on the southeastern segment of a ~2-km-long fracture field formed since 1998 on the eastern edge of the summit area^7^. Its cone formed on the lower eastern flank of the “old” SEC^15^ during 56 eruptions (mainly lava fountaining episodes) between 2007 and 2014, building up a cone ~300 m in height^16^,^7^. At a larger scale, it is worth noting that SEC, NSEC and numerous eruptive fissures opened along the high SE flank of the volcano between 1985 and 2006 following the same NW-SE structural trend. This system is the continuation in the summit area of a shallow (1–4 km) tectonic structure separating two sectors of Etna prone to flank instability and characterized by different kinematics^7^,^17^.

## Chronology of paroxysms and fracture opening in February 2013

**Figure 2:**
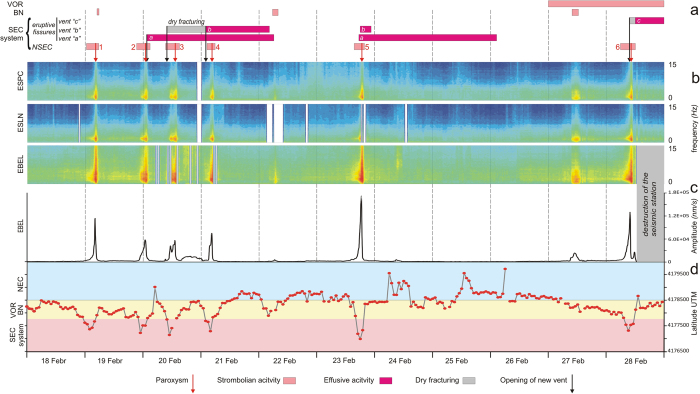
**a**) Eruptive activity, **b**) spectrograms of the seismic signal at ESPC, ESLN, EBEL, **c**) amplitude of volcanic tremor at EBEL, and **d**) latitude of the centroid of volcanic tremor from 18 to 28 February 2013. Coloured bands in d) mark the approximate latitude of the North-East Crater (NEC), Bocca Nuova (BN), Voragine (VOR) and South-East Crater (SEC) system.

Table 1 summarizes eruptive activity at the summit craters and seismic data recorded at EBEL in February 2013 (all times are UT). During the month, NSEC produced six short-lived (a few hours-long) paroxysmal eruptions. The first four episodes occurred between February 19 and 21 with repose periods lasting a few hours; the last two paroxysms were on February 23 and 28, respectively (Fig. 2). Two main stages of fracturing phenomena also took place at the southeastern base of NSEC on February 20 and 28.

A slow increase in the amplitude of background seismic radiation (so-called volcanic tremor) heralded the first paroxysmal eruptive activity, which started late at night on February 18 (Table 1). During this first paroxysm, weak Strombolian activity also occurred at BN. The tremor peaked at ~1.12 × 10^5^ nm/s during the climax of the lava fountain around 04:05 the day after (February 19, Fig. 2a,c). The amplitude of volcanic tremor was calculated from the root mean square (RMS) value of the seismic signal over consecutive 5-min intervals, considering the bottom 25% (25th percentile) of the RMS value in order to remove any undesired transient event (Fig. 2c).

The tremor gradually decreased in the morning of February 19, but increased again soon after 21:00 the same day, leading to another lava fountain within about 17 h. The second paroxysm began around midnight between February 19 and 20. It culminated at 00:50 with the opening of a new 600 m long eruptive fissure at the base of the NSEC that extended down to 2850 m above sea level (Vent “a” in Fig. 1). Vent “a” opened at 0.17 m/s in ~1 hour and fed a lava flow for almost 2½ half days.

**Figure 3:**
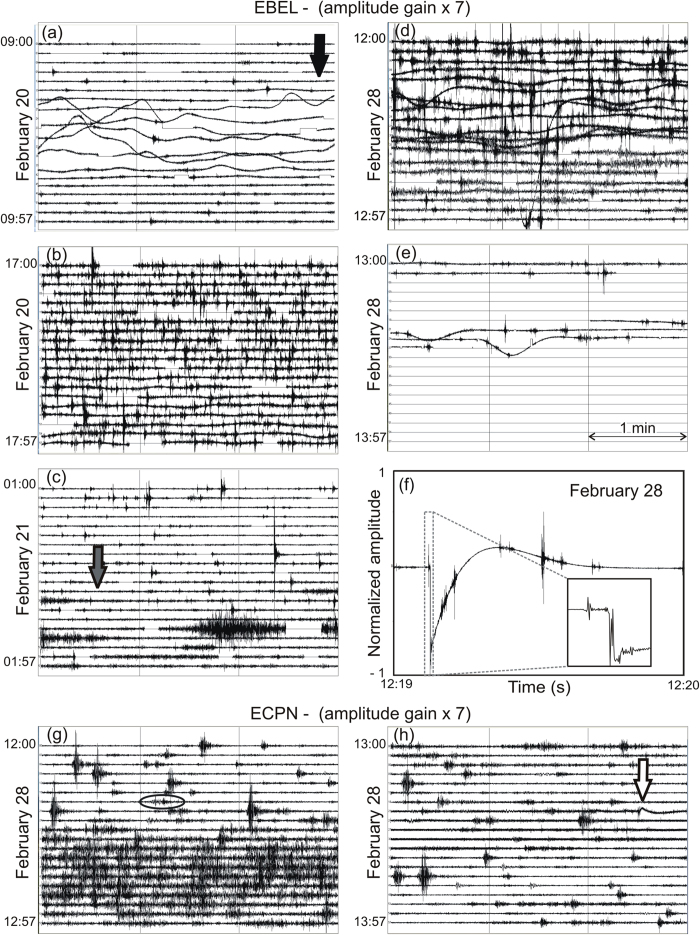
Seismic records at EBEL- (**a**–**f**) and ECPN- (**g**,**h**) vertical component. Seismograms (**a**–**e**) and (**g**,**h**) depict 3 min of signal per row, for a total of 1 h each. Arrows mark: the start of dislocation at EBEL (a, black), the activation of Vent “b” (c, grey), and the only evidence of dislocation at ECPN (h, white). The offset visible in the central part of the seismogram in (**d**) was associated with the seismic event that caused the strong jolt of EBEL at 12:19 on February 28. A 1-min excerpt (from 12:19 to 12:20) of this signal is shown in (**f**) along with a 1.2s zoom of its onset (see inset). The ellipse in (**g**) highlights the same event recorded at 12:19 at ECPN.

The first evidence of rock dislocation at EBEL occurred on February 20, about 6 h after the end of the second lava fountain. Between 09:14 and 09:44, the seismic signal underwent repeated low-frequency oscillations (Fig. 3a). The largest offset was of ~0.8 × 10^4^ nm/s. Towards the end of this phenomenon, the amplitude of volcanic tremor increased, heralding the third eruptive, paroxysmal activity shortly after (Table 1, Fig. 2). When the third paroxysm took place between 13:00 and 14:45, a dry (that is without magma emission) fracture field over a length of ~600 m and a width up to ~120 m opened in the area of Belvedere, close to EBEL (see the graben structure in Fig. 1a, b, partially overlapped by Vent “c” that would open later, on February 28). The fracture field was mainly characterized by NW-SE trending extension fractures with opening displacement (apertures) as great as 1.45 m, and normal faults with moderate vertical displacement (<1 m on the southern edge). The overall extension direction of these fractures (by measuring the shifts in the matching contact points at their walls) was ~NE-SW. Despite short-lived interruptions, data transmission from EBEL continued with minor, temporary offsets of the signal until 22:00 on February 20. The return of the seismic radiation to the background level after the third paroxysm was also punctuated by hundreds of short-lasting (4s on average) events per hour (Fig. 3b). This micro-seismicity continued the following day, abating only a couple of hours before the forth-paroxysmal episode.

On February 21, at 01:33, the lower portion of the fracture field generated a small lava flow (Vent “b”), which spread rapidly along the western wall of Valle del Bove (Fig. 1a, Table 1). Assuming the fracturing process covered a distance of ~950 m from the NSEC conduit over 979 minutes (from 09:14 on February 20), the dyke propagated at ∼0.02 m/s. The 1-h record (from 01:00 to 01:59) depicted in Fig. 3c highlights a few signals with spectral energy mostly between 2 and 8 Hz and maximum duration ~2 min. These signals were concurrent with landslides that followed the opening of the new vent, as documented by INGV video cameras. Immediately after the lava emission, there was the fourth eruptive paroxysm (Fig. 2, Table 1). On the morning of February 22, the vents “a” and “b” ceased to erupt, while Strombolian activity continued at the BN for a few hours.

In the evening on February 23, the amplitude of volcanic tremor peaked at 1.69 × 10^5^ nm/s, the highest value in the entire time span analysed (Fig. 2c). The increase of tremor accompanied the fifth paroxysm and the reactivation of the vents “a” and “b” (Table 1). Other modest buildups of volcanic tremor were concurrent with Strombolian activity at VOR and BN (Fig. 1a) on February 22 and 27-28, without any evident sign of ground fracturing either before or after volcanic activity. Micro-seismicity remained at low levels with a few isolated events during those days.

On February 27, Strombolian activity began at VOR and, for a few hours, at BN (Fig. 2a).

On the morning of February 28, the sixth eruptive paroxysm occurred along with the second period of ground fracturing. A 30s-long offset was recorded by EBEL at 11:57. Subsequent temporary offsets occurred over ~40 min of signal from 12:00 on. In this time span, micro-seismicity included a few high-amplitude events (Fig. 3c). The largest one struck EBEL at 12:19 (Fig. 3d,f), yielding an offset of 2.57 × 10^4^ nm/s, namely ~3.2 times greater than the largest offset recorded on February 20. By 12:25, a new NW-SE, discontinuous eruptive fissure opened from the SW flank of the NSEC to nearby the previously formed fracture field at an altitude of ~2940 m (Vent “c”, Fig. 1). Vent “c” opened at a distance of ~780 m from the NSEC 28 minutes after the onset of the ground fracturing process. In this case, the dyke intrusion achieved a speed of 0.46 m/s. The lava flow issuing from the lower segment of Vent “c” submerged and destroyed the seismic station EBEL shortly after. Seismic records stopped completely at 13:27 on February 28 (Fig. 3e).

## Observations at the permanent seismic network in February 2013

Neither micro-seismicity nor low-frequency displacements documented by EBEL seismograms on February 20 and 28 were recorded by the other seismic stations. The lack of these signals also holds for ECPN located ~1 km away from EBEL (Fig. 1c). Note that ECPN was out of order on February 20. The comparison between EBEL and ECPN highlights differences in their seismic records not only before, but also immediately after the major ground displacement occurring at EBEL (see Fig. 3d,e,g,h). In particular, on February 28 at 12:27, ECPN recorded a temporary increase in the tremor amplitude (Fig. 3g), which was also detected at the other stations except EBEL. This increase was concurrent with vigorous Strombolian activity following the sixth lava fountain (see Fig. 2a,b and Table 1). Furthermore, a low-frequency oscillation at 13:23 (white arrow in Fig. 3h) at ECPN had no match in the EBEL seismogram (Fig®. 3e).

Despite these differences, all stations detected variations in seismic energy radiation associated with the paroxysms (Fig. 2b,c). The centroid location of volcanic tremor changed shortly (a few hours) before each lava fountain episode. Based on the amplitude decay of seismic radiation at a minimum of 8 stations^18^, the 3D-location of the centroid moved within the volcano edifice from ~1 km below the summit craters NEC, VOR and BN to the surface nearby NSEC and Belvedere, throughout all impending paroxysms (Figs 1a,2d). This change, which followed a typical spatial evolution well described by ref. 19, was concurrent with the increase of amplitude of volcanic tremor in all of the seismic stations (Fig. 2a–c).

Throughout the paroxysms, the spectral content of the seismic signals changed too. The spectrograms in Fig. 2b span from 18 to 28 February 2013, and depict 5-min long successive spectra with resolution of 0.29 Hz. Seismic signals were corrected for the technical equipment response. The frequency peaked between 1.3 and 2 Hz at all stations in the climax of the six paroxysms. Also evident were: i) the gradual increase in the energy of seismic radiation a few hours before each paroxysmal activity, and ii) the sudden return to background levels at the conclusion of lava fountains (Fig. 2a,b). Apart from that, the spectrum was generally broader (with peaks up to ~8 Hz) at EBEL than at the other stations (Fig. 2b).

## Conceptual model

Surface faulting and displacement affected the region of Belvedere on Etna in February 2013. We may infer that the phenomenon affected a small volume of rock (≪1 km^3^), given that it was detected only at one seismic station. Fracturing activity was indeed associated with micro-seismicity recorded by EBEL only (Fig. 3). In addition, temperature and highly fractured material strongly affected the zone between NSEC and Belvedere, which was repeatedly cut by dry and eruptive NW-SE fissures in the recent past^5^. The low shear strength of volcaniclastic deposits at Etna is evident in the 3-D seismic tomography, in which relatively low values of the rate between P- and S-wave velocity (V_P_/V_S_ ≤ 1.64) characterize the first 2 km thickness of the volcanic pile above sea level in the central part of the volcano^20^. Analyses of basaltic samples in laboratory demonstrate that cracks account for up to nearly 90% of the difference in velocity with respect to unaltered solid material^21^. Temporal and spatial variations in the local stress conditions are to be expected in rapidly evolving magmatic systems^22^, while attenuation of seismic waves can also affect the volcanic environment notably^20^,^23^,^24^.

We believe that the fracturing affecting Belvedere (Fig. 1) originated from a shallow dyke that intruded during two distinct stages on February 20 and 28, respectively (see Fig. 3). Sited on the fracture field, EBEL provides exceptional evidence of the evolution of fracturing and intrusive processes near their source throughout both stages.

**Figure 4:**
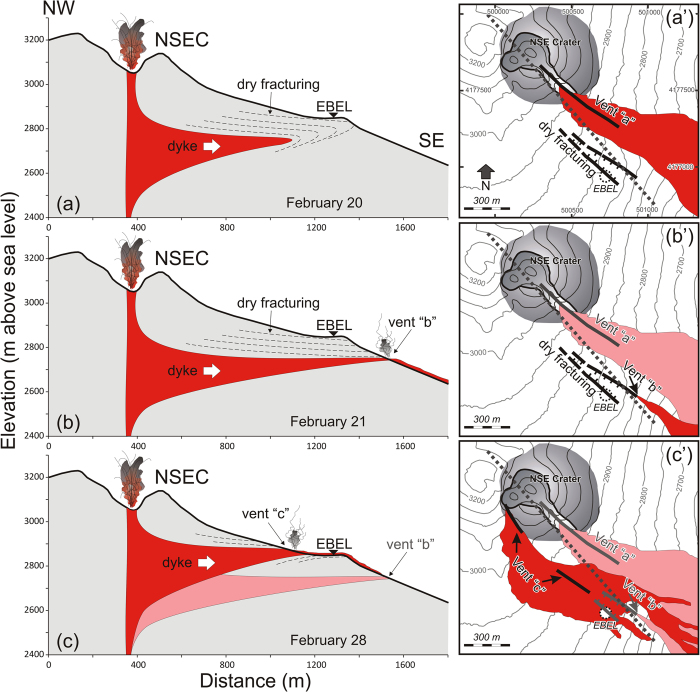
Cross sections (**a**–**c**) and map views (a’, c’, d’) illustrating the conceptual model of the main intrusion episodes occurred between 20 and 28 February 2013. The trace of the cross section corresponds to the NW-SE dashed grey line in a’), b’), c’) and in Fig. 1a. (**a**) dry fracturing close to EBEL station on February 20 (Vent “a” is not showed being out of perspective). (**b**) opening of Vent “b” and dry fracturing intensification on February 21. (**c**) opening of Vent “c” that issued the lava flow that destroyed EBEL on February 28. (a’), (b’), (c’) depict the opening of fractures and vent activation with the lava flows. Note that the graben structure in (c’) was totally buried by the lava flow of Vent “c”. The geographical coordinates in map (a’) are expressed in UTM projection, zone 33 N.

*February 20*, *Stage 1*. From the conduit of the NSEC (at depth of 400 m), a dyke propagated southeastward and intruded up to a few tens of meters from the surface (Fig. 4). A network of fractures opened above the tip of the dyke, creating a NW-SE small graben at the surface characterized by major displacement on its southern edge, in the Belvedere area (Fig. 1a,b). The limited size of the fracture field and the slight asymmetry of the graben support the hypothesis that the dyke was only a few tens/hundred meters below the slightly eastward sloping surface^6^.

From the seismic point of view, this first stage had visible effects at EBEL only (Fig. 3a). At first it was characterized by a low-frequency oscillation between 09:14 and 09:44 (black arrow in Fig. 3a), probably induced by the initial propagation of the dyke within the growing fracture field. After the third paroxysm occurred between 13:00 and 14:45, hundreds of short-lasting events accompanied the opening of the graben (Fig. 3b). The propagating dyke left the fractures “dry” in the Belvedere zone at altitude 2900 m, as the magma was drained at depth along the lower portion of the fracture. This means that the magma intruded a few tens of meters below EBEL seismic station, feeding eruptive activity a few hours later at the nearby Vent “b” at altitude 2750 m (Fig. 4b). The dyke intrusion (counted from the onset of fracturing to the activation of Vent “b”) was rather slow (0.02 m/s). Low-frequency oscillations registered at EBEL after ~17:35 are probably due to magma movements and pulses inside the fractures under the seismic station (Fig. 3b). Note also that the centroid location of volcanic tremor was within the volcano edifice at ~2 km above sea level; it migrated upward and laterally according to the previously described path followed by the dyke.

*February 28*. The second stage led to the climax of the fracturing at Belvedere. This time the dyke was shallower and thicker (presumably up to 1.9 m according to the average thickness of historical and recent dykes)^25^,^8^. The intrusion moved faster (0.46 m/s) than during the previous stage, and fed the effusive Vent “c” just upslope from EBEL (Fig. 4c), which underwent a strong jolt due to the breaking rocks close to the station (Fig. 3f). Evidence of ground fracturing marked by micro-seismicity was recorded at EBEL only, while ECPN merely recorded a small signal corresponding to the major fracturing event at 12:19 (see ellipse in Fig. 3g). This documents the proximity of the fracturing source to EBEL, which was finally overwhelmed by the lava flows at 13:27 on February 28. EBEL was certainly subjected to high temperatures and strong ground deformation, but most likely it stopped transmitting when the lava flows destroyed the data transmission systems.

An offset of the seismic signal, undetected at EBEL, was recorded by ECPN at 13:23 (white arrow in Fig. 3h). Since ECPN is at a higher altitude (3050 m above sea level) than EBEL (2899 m), we suppose that the offset was caused by the temporary propagation of its source towards ECPN. This explanation is compatible with a dyke propagating from the NSEC conduit towards the surface in a NW direction. Additional evidence of this final stage of the dyke intrusion comes from the temporary increase in the energy of seismic radiation recorded by all the seismic stations immediately after the rock fracturing at EBEL (Fig. 3c,f). Finally, magma reached the surface through a segment of the new fracture field feeding Vent “c” (Fig. 1a, Fig. 3).

These observations shed light on the development of a shallow dyke emplacement at Etna, identifying a kind of seismic footprint related to magmatic intrusions and faulting that may form the basis for recognizing similar processes in other well monitored basaltic volcanoes worldwide, such as Stromboli in Italy^11^; Kilauea in Hawaii^10^; Krafla and Bardarbunga in Iceland^26^,^27^.

## Additional Information

**How to cite this article**: Falsaperla, S. and Neri, M. Seismic footprints of shallow dyke propagation at Etna, Italy. *Sci. Rep.* **5**, 11908; doi: 10.1038/srep11908 (2015).

## Figures and Tables

**Table 1:** Chronology of volcanic activity and opening and evolution of fracture fields.

Observation	Crater - Location	Date (dd/mm/yyyy)	Onset (hh:mm)	Climax (hh:mm)	End (hh:mm)	Max. tremor amplitude (nm/s)	Information
*Lava fountaining*	NSEC	19/02/2013	03:45	04:05	05:00	1.12E + 05	Seismic data
Mild Strombolian activity	BN	19/02/2013	04:30		05:30		Visual observations
*Lava fountaining*	NSEC	20/02/2013	00:15	00:50	01:50	5.71E + 04	Seismic data
Vent “a” opening and activation	3000-2850 m a.s.l.	20/02/2013	01:15				Visual observations
Graben opening	Belvedere	20/02/2013	09:14				Seismic data
*Lava fountaining*	NSEC	20/02/2013	11:00	13:10	13:50	5.56E + 04	Seismic data
Vent “b” activation	2800 m a.s.l., Valle del Bove	21/02/2013	01:33				Visual observations
*Lava fountaining*	NSEC	21/02/2013	03:50	04:30	05:05	7.40E + 04	Seismic data
Vent “b” ceased to erupt	Belvedere	22/02/2013			05:30		Visual observations
Vent “a” ceased to erupt	3000-2850 m a.s.l.	22/02/2013			07:00		Visual observations
Mild Strombolian activity	BN	22/02/2013	06:00		09:30		Visual observations
*Lava fountaining*	NSEC	23/02/2013	17:55	18:35	19:25	1.69E + 05	Seismic data
Vent “a” resumed erupting	3000-2850 m a.s.l.	23/02/2013	18:30				Visual observations
Vent “b” resumed erupting	Belvedere	23/02/2013	18:40		22:00		Visual observations
Mild Strombolian activity	BN	27/02/2013	00:00				Visual observations
Mild Strombolian activity	VOR	27/02/2013	09:00		11:00		Visual observations
*Lava fountaining*	NSEC	28/02/2013	09:30	10:10	11:00	1.28E + 05	Seismic data
Vent “c” opening (dry fractures)	Belvedere	28/02/2013	11:57		12:25		Seismic data
Vent “c” activation	2940 m a.s.l., Belvedere	28/02/2013	12:25				Visual observations
Breakdown of EBEL station	Belvedere	28/02/2013			13:27		Seismic data
Vent “c” ceased to erupt	2940 m a.s.l., Belvedere	01/03/2013			11:00		Visual observations
